# Foreign Notes and Excerpta

**Published:** 1868-08-01

**Authors:** 


### *FOREIGN NOTES AND EXCERPTA.*

M. Guibort lately brought before the Medical Society of the Hospital of Paris, a man fifty years old, who had passed seven iron rings over his penis towards the pubes. The result was severe constriction, followed by apparent gangrene. M. Richard attempted in vain to cut the rings with pliers, and a subsequent attempt with a clockmaker's saw was successful in cutting two of the rings after the destruction of six or seven saws. M. Mathien, a skillful surgical intrument maker, by means of the most powerful pincers, saws and pliers succeeded in removing the remaining five. The gangrenous inflammation was arrested, and the organ saved.

In a similar case recorded by Natalis Guillot, a gold wedding ring was used for a similar purpose. In this case the part was plunged in liquid mercury, which dissolved the ring.—*Lancet.*

---

A case of *Ectropia Vesicæ* has been exhibited to the Clinical Society of London. The vagina could be traced and the uterus could be felt through the rectum. The labiæ were fully, but the nymphæ only slightly developed; the pubes cleft, and the abdomen deficient in the middle line from the umbilicus downwards. In this case the bladder was covered with a structure resembling skin, as low as the level of the ureters, and this surface did not cause trouble from its irritability.—*Lancet.*

---

M. Gubler, Professor of the Faculty of Medicine of the Hospital Beaujon (Paris), and Vice-President of the Therapeutical Society, in a review of the French Codex, asserts that *Albumen* plays a much more important part in therapeutics than has been hitherto assigned to it. That in relatively large doses it becomes a solvent for substance considered insoluble, and even for those, which, in other proportions coagulate it firmly. Moreover, that the substances thus dissolved lose at the same time some of their chemical properties, and the reactions which they ordinarily occasion are thenceforward prevented.

This fact is susceptible of numerous and various applications in therapeutics. Not only should albumen be prescribed rigorously in every form containing coagulable substances, but if it is desired to produce general or diffused effects, it will often be advantageous to prepare beforehand albuminous solutions of the active principles with the precaution demanded for these delicate preparations. As albumen dissolves iron, it is proposed to administer this metal in a state of minute division. The process might be extended to many metallic preparations, with which albumen might be charged, whenever their general and alterative effects are desired. In solution, in the form of albuminates, iron, manganese, copper, mercury and silver, would exercise no injurious influence upon the *primæ viæ*, would penetrate the circulation more readily, and would present at once, as it were, a first degree of assimilation.

---

Copaiba — M. Gubler states, is a compound of volatile oil and of copahivic acid, which is a resin. The essential oil is eliminated by the lungs, and the resin by the kidneys, hence in the treatment of blenorrhagia, if copaiba, deprived of its essential oil be administered, the efficiency of the drug will not be dimidished, and the patient be relieved from that horrible breath which betrays it.

---

About the Absorption of Medicines.—M. Demarquay remarks that the time included in experiments upon absorption should be divided into four periods. 1st. That occupied by the drug in reaching the absorbent organs. 2d. That by its retention in the blood. 3d. That consumed in its elimination, and—4th (in the case of drugs eliminated by the kidneys). That of its retention in the bladder.

These experiments give only a distant approximation to the period extending between the administration of the drug and its absorption. M. Demarquay confined his experiments to *Iodide of Potassium*, and determined that after having been administered by the mouth, it was eliminated by the kidneys in from nine to fifteen minutes; but this period was not constant, for in some of the experiments the drugs could not be recovered at all. The same dose administered by the rectum exhibits itself in the urine much more constantly in from two to seven minutes. The drug introduced through the mucous surface of the bronchi in antomized water could be detected in the saliva in five to six minutes.

After sixteen injections of the iodide into the bladder, eight times the drug could not be recovered, and in the other eight cases it appeared in the saliva in from thirty five minutes to six hours after the injection.

The salt, when in solution, was absorbed but feebly by the skin, when quite actively where applied in a pomade.

---

Puerperal Convulsions.—Dr. T. C. Osborn, Greensboro, reports in the July number (1868) of the *New Orleans Journal of Medicine*, a case of recovery from "Eclampsia Bimiparturientis," under the use of chloroform, the forceps and morphia hypodermically. "Protests against indiscriminate bleeding," anterior to delivery.

Cæsarian Section.—D. Warren Brickell, Professor of Obstetrics in the New Orleans School of Medicine, reports in the same journal a successful (to both mother and child) case of this operation, after a labor of ten days' duration, the necessity of which originated in friability of the tissues of uterus and vaginal adhesion, nothing abnormal being observed in the bones. The novelty in the operation was the necessity of closing the uterine incision with sutures (silver wire), which were permitted to remain, and after five months had occasioned no inconvenience. The entire wound healed by first intention.

